# ﻿First record of the genus *Trilacuna* Tong & Li, 2007 (Araneae, Oonopidae) from Xizang, China, with descriptions of three new species and one newly recorded species

**DOI:** 10.3897/zookeys.1229.145844

**Published:** 2025-02-27

**Authors:** Songlu Shi, Dongju Bian, Yanfeng Tong, Shuqiang Li

**Affiliations:** 1 College of Life Science, Shenyang Normal University, Shenyang 110034, Liaoning, China Shenyang Normal University Shenyang China; 2 Key Laboratory of Forest Ecology and Management, Institute of Applied Ecology, Chinese Academy of Sciences, Shenyang 110016, China Institute of Applied Ecology, Chinese Academy of Sciences Shenyang China; 3 Institute of Zoology, Chinese Academy of Sciences, Beijing 100101, China Institute of Zoology, Chinese Academy of sciences Beijing China

**Keywords:** Biodiversity, distribution, goblin spiders, morphology, new record, taxonomy, Tibet

## Abstract

Four species of the genus *Trilacuna* Tong & Li, 2007 from Xizang, China are recognized, including three new species and one newly recorded species: *T.bangla* Grismado & Ramírez, 2014, *T.mainling* Tong & Li, **sp. nov.** (♂♀), *T.metok* Tong & Li, **sp. nov.** (♂♀) and *T.zayu* Tong & Li, **sp. nov.** (♂♀). Descriptions, diagnoses and photomicroscopy images are provided.

## ﻿Introduction

The family Oonopidae Simon, 1890, also known as goblin spiders, are composed of tiny spiders between 0.5 and 3 mm. Oonopidae is among the nine most diverse spider families with 1962 extant described species in 115 genera (WSC 2025).

**Figure 1.**
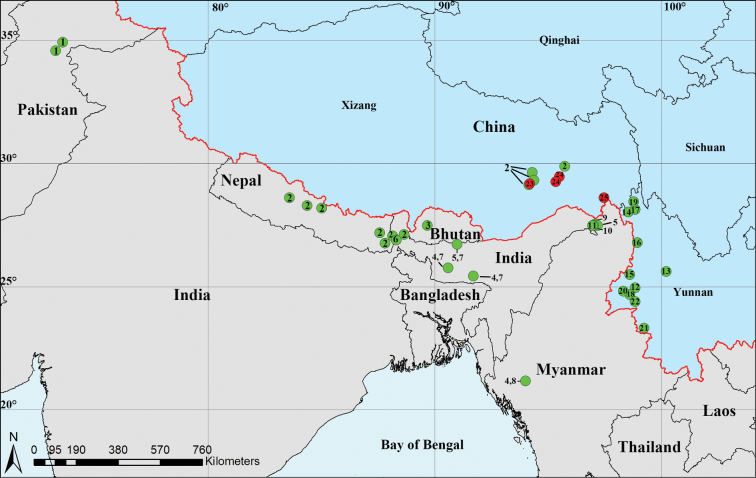
Distribution records of *Trilacuna* species from the Himalaya region: green circles represent 22 known species, red circles indicate the three new species. 1. *T.hazara*; 2. *T.bangla*; 3. *T.aenobarba*; 4. *T.besucheti*; 5. *T.loebli*; 6. *T.mahanadi*; 7. *T.meghalaya*; 8. *T.changzi*; 9. *T.hponkanrazi*; 10. *T.triseta*; 11. *T.zhigangi*; 12. *T.bawan*; 13. *T.cangshan*; 14. *T.cuneata*; 15. *T.datang*; 16. *T.fugong*; 17. *T.gongshan*; 18. *T.longling*; 19. *T.rastrum*; 20. *T.wuhe*; 21. *T.wumanshan*; 22. *T.xiaoheishan*; 23. *T.mainling* sp. nov.; 24. *T.metok* sp. nov.; 25. *T.zayu* sp. nov.

The genus *Trilacuna* Tong & Li, 2007 currently comprises 48 species known from Iran to the Korean Peninsula and south to Sumatra, Indonesia (Ma et al. 2024). *Trilacuna* has the highest species diversity in the Himalaya region: 22 species have been recorded there (Fig. 1), including one species from Pakistan, one species from Bhutan, one species from Nepal, five species from India, six species from Myanmar and 11 species from northwestern Yunnan, China (Tong and Li 2007; Grismado et al. 2014; Liu et al. 2019; Tong et al. 2019, 2020; Ma et al. 2023). At the same time, there are no records of this genus from Xizang.

In this paper, *Trilacuna* is recorded for the first time from Xizang Autonomous Region and three new species and one newly recorded species of the genus are described and photographed.

## ﻿Material and methods

The specimens were examined using a Leica M205 C stereomicroscope. Fine details were studied under an Olympus BX51 compound microscope. Endogynes were cleared in lactic acid. Photomicroscope images were taken with a Canon EOS 750D zoom digital camera (24.2 megapixels) mounted on the Olympus BX51. Raw photos were first stacked with Helicon Focus v. 8.2.0 to get the composite images, which were then processed in Adobe Photoshop CC 2020. Scanning electron microscope images (SEM) were taken under high vacuum with a Hitachi S-4800 after critical-point drying and gold-palladium coating. The distribution map was generated with ArcGIS v. 10.2 (ESRI Inc.). All measurements were taken using the Olympus BX51 and are in millimeters. Taxonomic descriptions follow Tong et al. (2020). Type material is deposited in the Shenyang Normal University (SYNU) in Shenyang, Liaoning Province, China (curator: Yanfeng Tong).

The following abbreviation is used in the text: **ALE** = anterior lateral eyes.

## ﻿Taxonomy

**Family Oonopidae Simon, 1890**

### ﻿Genus Trilacuna

Taxon classificationAnimaliaAraneaeOonopidae

Tong & Li, 2007

44B6743B-59B0-52AB-844E-77702FC87429

#### Type species.

*Trilacunarastrum* Tong & Li, 2007 from Yunnan, China.

#### Diagnosis.

See Tong et al. (2020).

#### Composition.

Fifty-one species, including three described here.

#### Distribution.

From Iran to the Korean Peninsula and south to Sumatra, Indonesia.

### ﻿ Trilacuna bangla

Taxon classificationAnimaliaAraneaeOonopidae

Grismado & Ramírez, 2014

C93C1B08-116C-52A7-93C6-CCEBD227D53D

**Figure 2.**
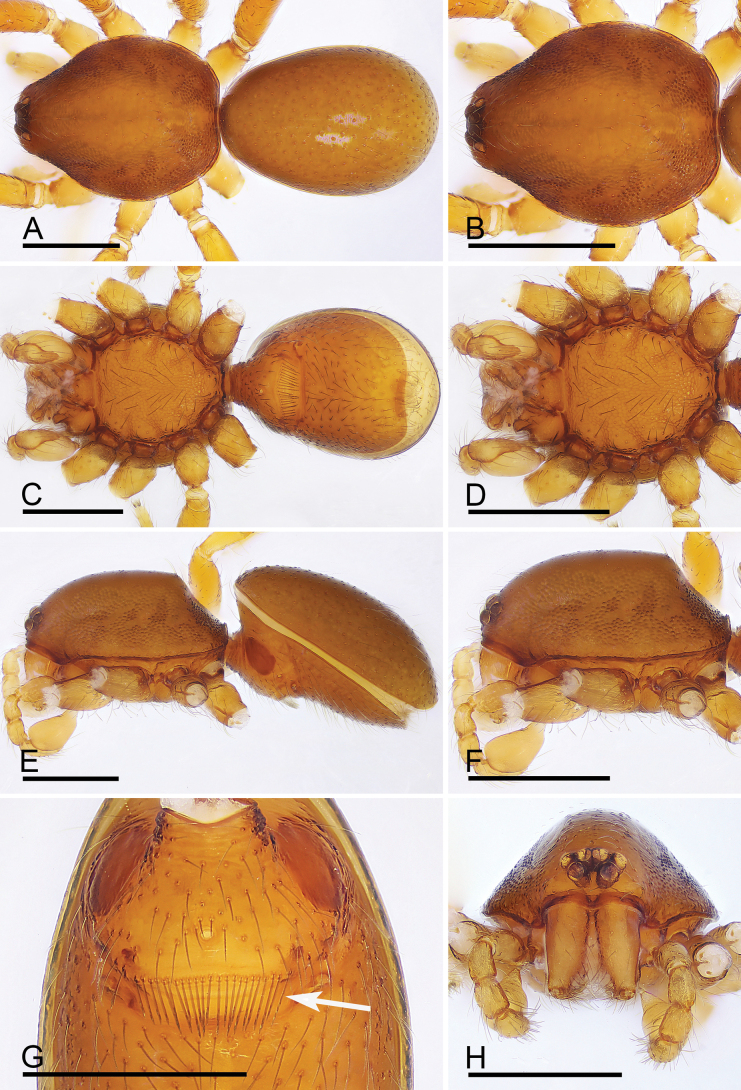
*Trilacunabangla* Grismado & Ramírez, 2014, male **A, C, E** habitus, dorsal, ventral and lateral views **B, D, F, H** prosoma, dorsal, ventral, lateral and anterior views **G** abdomen, ventral view, arrow shows the row of setae. Scale bars: 0.4 mm.

**Figure 3.**
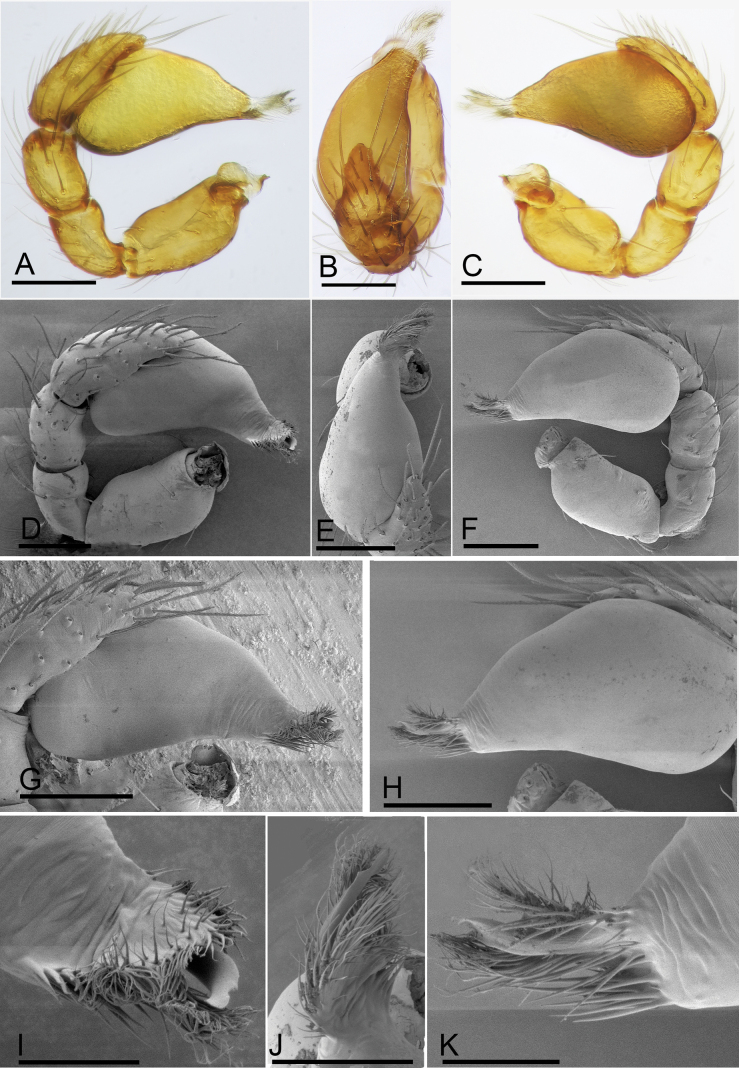
*Trilacunabangla* Grismado & Ramírez, 2014, male left palp **A–C** (light) and **D–K** (SEM) **A, D** prolateral view **B, E** dorsal view **C, F** retrolateral view **G** bulb, prolateral view **H** bulb, retrolateral view **I, J, K** distal part of bulb, prolateral, dorsal and retrolateral views. Scale bars: 0.1 mm (**A–G**); 0.05 mm (**I–K**).

**Figure 4.**
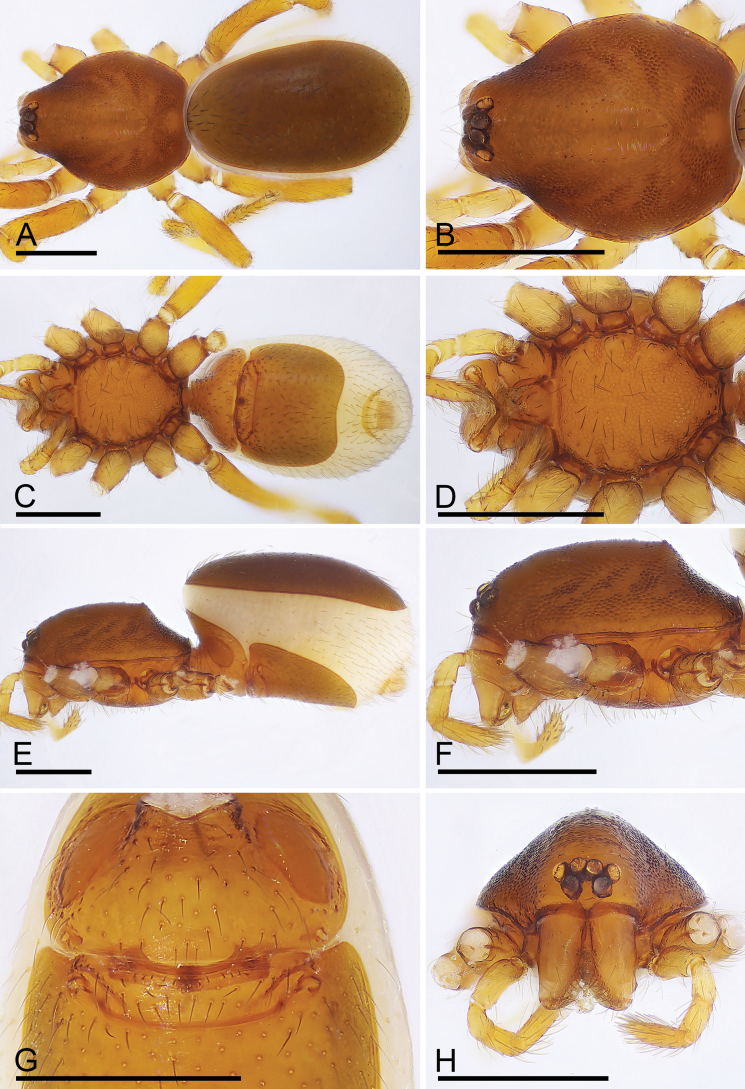
*Trilacunabangla* Grismado & Ramírez, 2014, female **A, C, E** habitus, dorsal, ventral and lateral views **B, D, F, H** prosoma, dorsal, ventral, lateral and anterior views **G** abdomen, ventral view. Scale bars: 0.4 mm.

**Figure 5.**
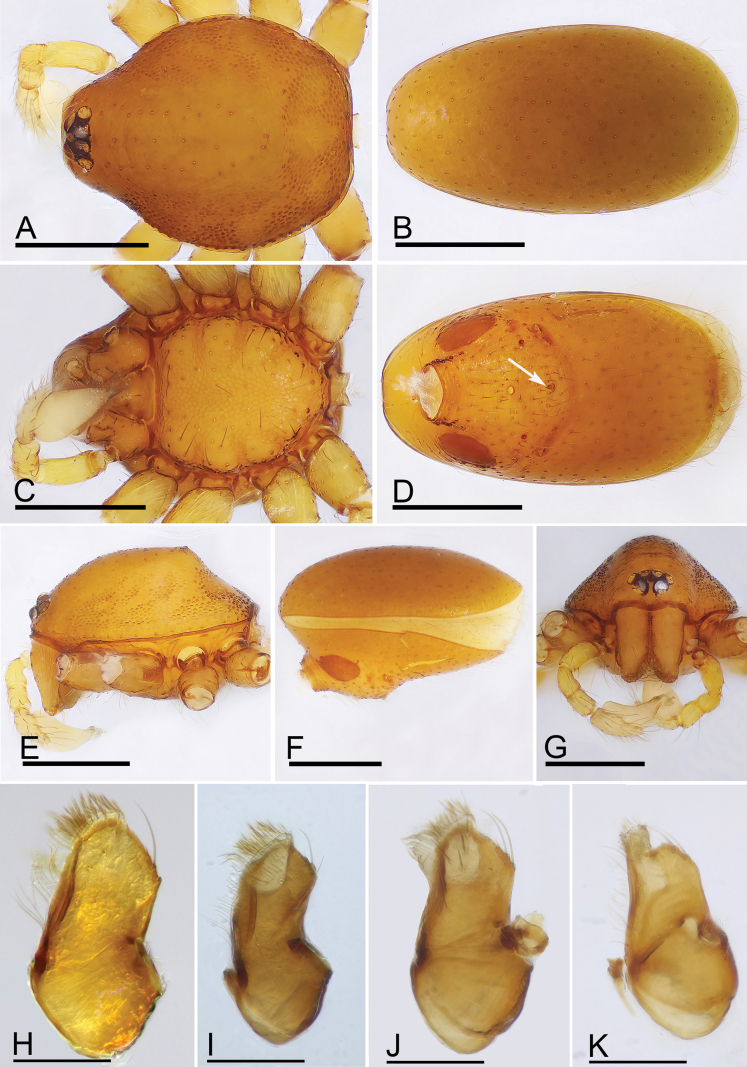
*Trilacunamainling* sp. nov., male holotype (**A–H**), *Trilacunametok* sp. nov. (**I**), *Trilacunabangla* (**J**), *Trilacunazayu* sp. nov. (**K**) **A, C, E, G** prosoma, dorsal, ventral, lateral and anterior views **B, D, F** abdomen, dorsal, ventral and lateral views, arrow shows the small wedge-shaped process **H–K** endites, ventral view. Scale bars: 0.4 mm.

Figs 2 , 3 , 4 , 5J , 14E, F

Trilacuna bangla Grismado & Ramírez, in Grismado et al. 2014: 57, figs 42A–F, 43A–N, 44A–H, 45A–I, 46A–T, 47A–L, 48A, C, E, 49A–H, 50A–F, 51A–E.

#### Material examined.

China • 1♂1♀ (SYNU-916–917); Xizang, Nyingchi City, N of Bayi Town; 29°38.24'N, 94°22.19'E, 2900 m elev.; 10.VII.2013; L. Lin leg. • 2♂2♀ (SYNU-918–921); S of Mainling Co.; 29°12.316'N, 94°12.649'E, 3060 m elev.; 13.VIII.2013; Q. Cao leg. • 1♂1♀ (SYNU-922–923); Mainling Co., Nanyi Town, Zhagonggou Scenic Area; 29°09.315'N, 94°12.869'E, 3153 m elev.; 28.VII.2012; Z. Yao & Z. Zhao leg.

#### Other material.

China • 2♂ (SYNU-F-933–934); Nyingchi City, N of Bayi Town; 29°38.24'N, 94°22.19'E, 2900 m elev.; 10.VII.2013; L. Lin leg. • 1♂ (SYNU-F-937); same data as above • 2♂ (SYNU-F-935–936); Bayi Town; 29°39.831'N, 94°20.669'E, 2990 m elev.; 12.VII.2013; L. Lin leg. • 1♀ (SYNU-F-938); Mainling Co.; 29°22.068'N, 94°24.663'E, 2965 m elev.; 11.VIII.2014; L. Lin leg. • 2♂1♀ (SYNU-F-939–941); S of Mainling Co.; 29°12.316'N, 94°12.649'E, 3060 m elev.; 13.VIII.2013; L. Lin leg. • 1♀ (SYNU-F-942); Mainling Co., Nanyi Town, Zhagonggou Scenic Area; 29°09.315'N, 94°12.869'E, 3153 m elev.; 28.VII.2012; Z. Yao & Z. Zhao leg. • 2♂ (SYNU-F-943–944); N of Mainling Co.; 29°13.310'N, 94°13.309'E, 3050 m elev.; 13.VIII.2013; Q. Cao leg. • 1♀ (SYNU-F-945); same data as above • 3♂3♀ (SYNU-F-948–953); Bomi Co., Zhamo Rd., nr Baqiong Vill.; 29°52.194'N, 95°43.505'E, 2880 m elev.; 19.VII.2013; L. Lin leg. • 1♂ (SYNU-F-954); Mainling Co.; 3.VII.1997; M. Wu leg.

#### Diagnosis and description.

See Grismado et al. (2014).

#### Variation.

Male epigastric area with a patch of enlarged setae in the specimens from India (Grismado et al. 2014: figs 47A, B, 51B), but the setae are in a regular row in the specimens from Xizang, China (Fig. 2C, G).

#### Distribution.

China (Xizang), India (West Bengal), Nepal (Fig. 1).

### ﻿ Trilacuna mainling

Taxon classificationAnimaliaAraneaeOonopidae

Tong & Li sp. nov.

10C194EB-8523-5728-AB8A-6300B114E3E7

https://zoobank.org/BD584F02-7DB4-4A08-93F8-C2A92BDD109D

**Figure 6.**
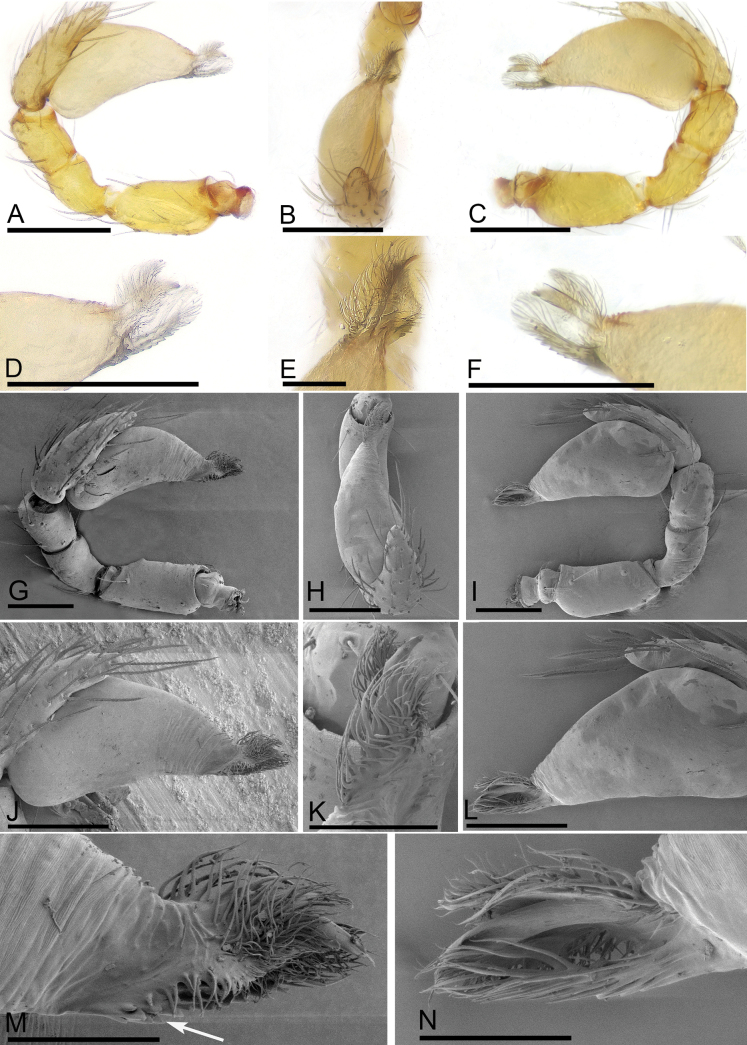
*Trilacunamainling* sp. nov., male left palp **A–F** (light) and **G–N** (SEM) **A, G** prolateral view **B, H** dorsal view **C, I** retrolateral view **D, M** distal part of bulb, prolateral view, arrow shows the serrated structure **E, K** distal part of bulb, dorsal view **F, N** distal part of bulb, retrolateral view **J, L** bulb, prolateral and retrolateral views. Scale bars: 0.1 mm (**A–C, G–J, L**); 0.05 mm (**D–F, K, M, N**).

**Figure 7.**
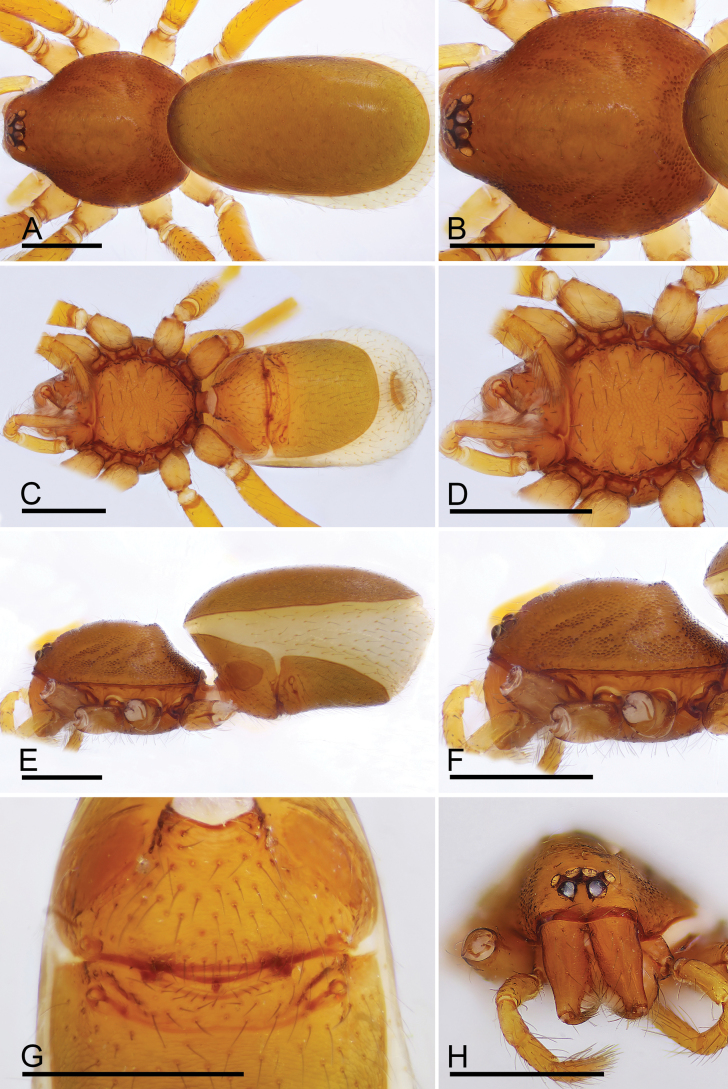
*Trilacunamainling* sp. nov., female paratype **A, C, E** habitus, dorsal, ventral and lateral views **B, D, F, H** prosoma, dorsal, ventral, lateral and anterior views **G** abdomen, ventral view. Scale bars: 0.4 mm.

Figs 5A–H , 6 , 7 , 14A, B

#### Material examined.

***Holotype*** China • ♂ (SYNU-914); Xizang, N of Mainling Co.; 29°13.310'N, 94°13.309'E, 3050 m elev.; 13.VIII.2013; Q. Cao leg. ***Paratype*.** China • 1♀ (SYNU-915); same data as holotype.

#### Etymology.

The specific name is a noun in apposition taken from the type locality.

#### Diagnosis.

The new species is similar to *T.bangla* in the shape of the bulb but can be distinguished by the male epigastric region with a small wedge-shaped process (Fig. 5D, arrow) vs. lacking, but with a row of enlarged setae (Fig. 2G, arrow); the psembolus with serrated structure at the base (Fig. 6M, arrow) vs. lacking, instead of cluster of fibers (Fig. 3I); the reticulated abdominal scutum of the female (Fig. 7A, C, E) vs. smooth (Fig. 4A, C, E); and the very small globular structure of the endogyne (Fig. 14B) vs. very large (Fig. 14F).

#### Description.

**Male.** Body: yellow, legs lighter; habitus as in Fig. 5A–F; body length 1.59. Carapace (Fig. 5A, E): 0.87 long, 0.72 wide; sides granulate. Eyes (Fig. 5A, G): well developed; ALE separated from edge of carapace by one diameter. Mouthparts (Fig. 5C, G, H): endites slender, distally not branched. Sternum (Fig. 5C): surface reticulated. Abdomen: 0.63 long, 0.48 wide; sperm pore situated at level of anterior spiracles; apodemes present, posterior spiracles connected by groove; with small wedge-shaped process situated between anterior and posterior spiracles; epigastric region slightly elevated (Fig. 5D, F). Palp (Fig. 6A–N): orange; 0.68 long (0.21, 0.12, 0.11, 0.24); femur not enlarged (width/length = 0.46); bulb triangle; psembolus with several rows of serrated structure at base, psembolus proper surrounded by numerous fiber structures.

**Female (*paratype*, SYNU-915).** As in male except as noted. Body: habitus as in Fig. 7A, C, E; body length 2.19. Carapace: 0.97 long, 0.79 wide; Abdomen: 1.37 long, 0.74 wide; dorsal and ventral scutum surface reticulated. Epigastric area (Figs 7G, 14A): with recurved, strongly sclerotized arches (sar). Endogyne (Fig. 14B): with narrow, transverse sclerite (tsc); with anterior large T-shaped sclerite (as) and posterior small globular structure (glo); transverse bars (tba) with pair of lateral apodemes (ap).

#### Distribution.

Known only from the type locality (Fig. 1).

### ﻿ Trilacuna metok

Taxon classificationAnimaliaAraneaeOonopidae

Tong & Li sp. nov.

641F91A2-9E3F-52D5-A9F7-8E846222FE40

https://zoobank.org/B4BA31AD-FDE2-4C1C-B992-D9E585E6CCF6

**Figure 8.**
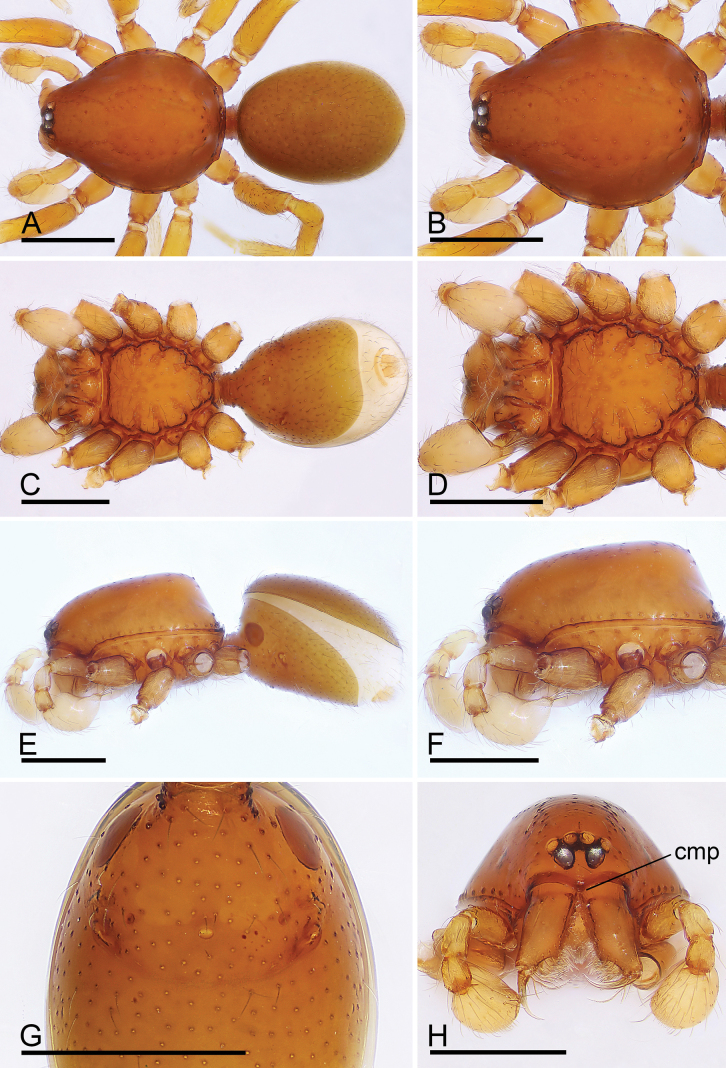
*Trilacunametok* sp. nov., male holotype **A, C, E** habitus, dorsal, ventral and lateral views **B, D, F, H** prosoma, dorsal, ventral, lateral and anterior views **G** abdomen, ventral view. Abbreviation: cmp = clypeus median projection. Scale bars: 0.4 mm.

**Figure 9.**
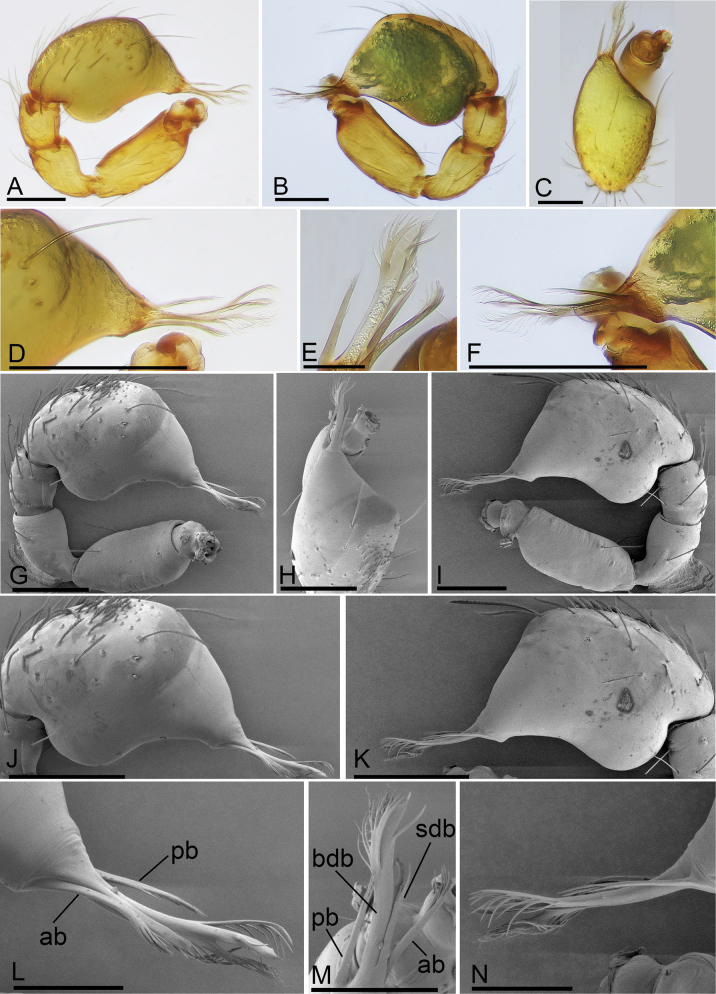
*Trilacunametok* sp. nov., male left palp **A–F** (light) and **G–N** (SEM) **A, G** prolatral view **B, I** retrolateral view **C, H** dorsal view **D, L** distal part of cymbiobulb, prolateral view **E, M** distal part of cymbiobulb, dorsal views **F, N** distal part of cymbiobulb, retrolateral view **J, K** cymbiobulb, prolateral and retrolateral views. Abbreviations: ab = anterior branch; bdb = broad dorsal branch; pb = posterior branch; sdb = slender dorsal branch. Scale bars: 0.1 mm (**A–K**); 0.05 mm (**L–N**).

**Figure 10.**
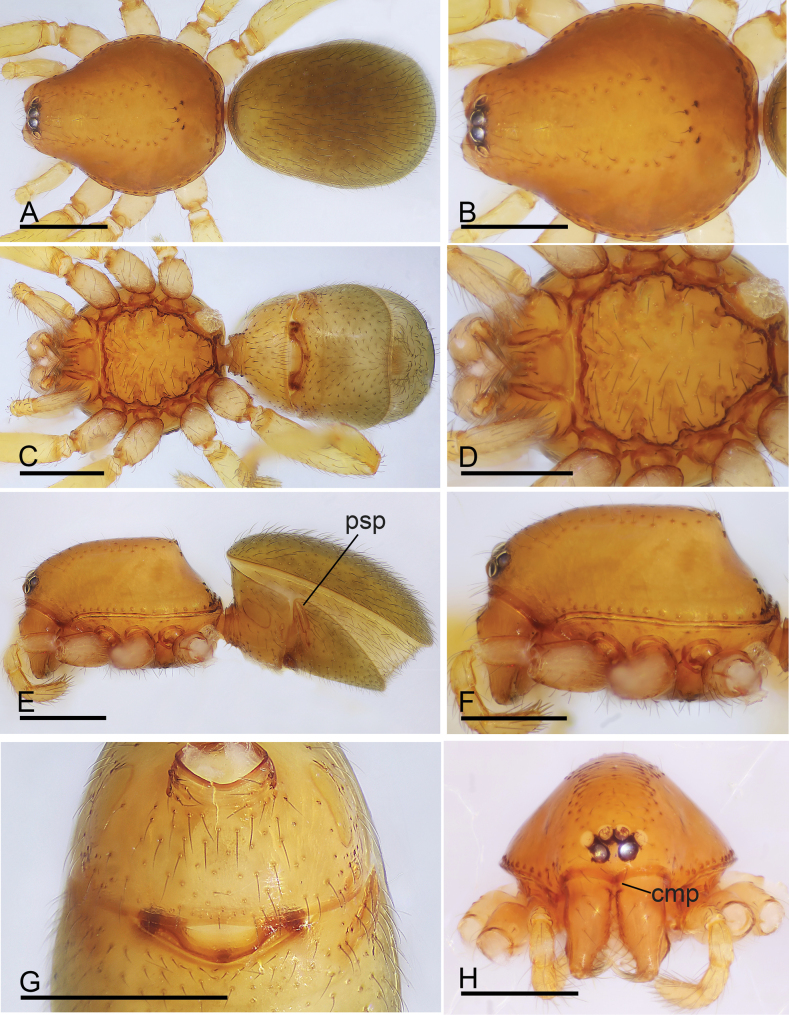
*Trilacunametok* sp. nov., female paratype **A, C, E** habitus, dorsal, ventral and lateral views **B, D, F, H** prosoma, dorsal, ventral, lateral and anterior views **G** abdomen, ventral view. Abbreviations: cmp = clypeus median projection; psp = posterior spiracles. Scale bars: 0.4 mm.

Figs 5I , 8 , 9 , 10 , 14C, D

#### Material examined.

***Holotype*** China • ♂ (SYNU-911), Xizang, Nyingchi City, Metok Co., nr Yadong Vill.; 29°20.605'N, 95°20.807'E, 1360 m elev.; 6.VIII.2013; L. Lin leg. ***Paratypes*.** China • 1♂1♀ (SYNU-912–913); same data as holotype • 1♀ (SYNU-924); same data as holotype.

#### Other material.

China • 1♂1♀ (SYNU-F-955–956); same data as holotype • 1♀ (SYNU-F-967); same data as holotype • 1♂2♀ (SYNU-F-976–978); same data as holotype • 1♀ (SYNU-F-963); near Metok Co.; 29°19.399'N, 95°20.448'E, 1300 m elev.; 3.VIII.2013; L. Lin leg. • 1♂1♀ (SYNU-F-973–974); same data as above • 1♂2♀ (SYNU-F-964–966); near Metok Co.; 29°19.382'N, 95°19.016'E, 980 m elev.; 2.VIII.2013; L. Lin leg. • 1♂2♀ (SYNU-F-957–959); Metok Co., hills nr Nongjiale; 29°19.087'N, 95°18.876'E, 1280 m elev.; 4.VIII.2013; Q. Cao leg. • 3♂ (SYNU-F-960–962); same data as above • 1♀ (SYNU-F-968); same data as above • 1♀ (SYNU-F-975); same data as above • 4♂ (SYNU-F-969–972); 108 km along highway from Bomi Co. to Metok Co.; 29°29.178'N, 95°26.868E, 1360 m elev.; 9.VIII.2013; L. Lin leg.

#### Etymology.

The specific name is a noun in apposition taken from the type locality.

#### Diagnosis.

Males of this new species are similar to those of *T.triseta* Tong & Li, 2000 in the branches of psembolus, but can be distinguished by the fused cymbium and bulb (Fig. 9A–N) vs. cymbium well separated (Tong et al. 2020: fig. 12A, B) and the coxae IV connected by radial furrow (Fig. 8D) vs. not connected (Tong et al. 2020: fig. 11F). Females of the new species can be distinguished from all congeners by the long, narrow posterior spiracles, which are situated at the anterior corner of the postgastric scutum (Fig. 10C, E) vs. normal size, far away the anterior corner (e.g., Figs 4G, 7G, 13G).

#### Description.

**Male.** Body: reddish-brown, legs yellow; habitus as in Fig. 8A, C, E; body length 1.61. Carapace (Fig. 8B, F): 0.81 long, 0.63 wide; sides smooth. Eyes (Fig. 8B, H): eyes well developed; ALE separated from edge of carapace by 1.25 diameters. Mouthparts (Figs 8D, H, 5I): labium distally not branched, with large membranous area at anterior part. Sternum (Fig. 8D): surface smooth, with large seta pits; with radial furrows between coxae I–II, II–III, III–IV and IV–IV. Abdomen (Fig. 8G): 0.87 long, 0.55 wide; surface smooth; sperm pore situated between anterior and posterior spiracles; apodemes present, posterior spiracles connected by groove. Palp (Fig. 9A–N): orange; 0.58 long (0.16, 0.13, 0.08, 0.21); femur elongated (width/length = 0.48); bulb and cymbium fused; psembolus including slender anterior branch (ab), slender dorsal branch (sdb), broad dorsal branch (bdb), and slender posterior branch (pb).

**Female (*paratype*, SYNU-913).** As in male except as noted. Body: habitus as in Fig. 10A, C, E; body length 2.19. Carapace: 0.97 long, 0.79 wide. Abdomen: 1.37 long, 0.74 wide; posterior spiracles narrow and long (psp), situated at anterior corner of postgastric scutum. Epigastric area (Figs 10G, 14C): without recurved, strongly sclerotized arches. Endogyne (Fig. 14D): with narrow, transverse sclerite (tsc); with an anterior large T-shaped sclerite (as) and a posterior small globular structure (glo); transverse bars (tba) with pair of strongly curved, thick lateral apodemes (ap).

#### Comment.

All the known species of the genus *Trilacuna* have a well-separated cymbium and bulb; the fused cymbium and bulb demonstrate that *T.metok* sp. nov. is quite different from all other species of *Trilacuna*. But based on the psembolus and somatic characters, e.g., the deeply incised labium and the large setae base on the male chelicerae, it is reasonable to consider this species in *Trilacuna*.

#### Distribution.

Known only from the type locality (Fig. 1).

### ﻿ Trilacuna zayu

Taxon classificationAnimaliaAraneaeOonopidae

Tong & Li sp. nov.

637D98E6-02BC-57CC-952D-C91AEBCD039E

https://zoobank.org/F7E44F6F-DF7E-4B16-B249-90D6354E7068

**Figure 11.**
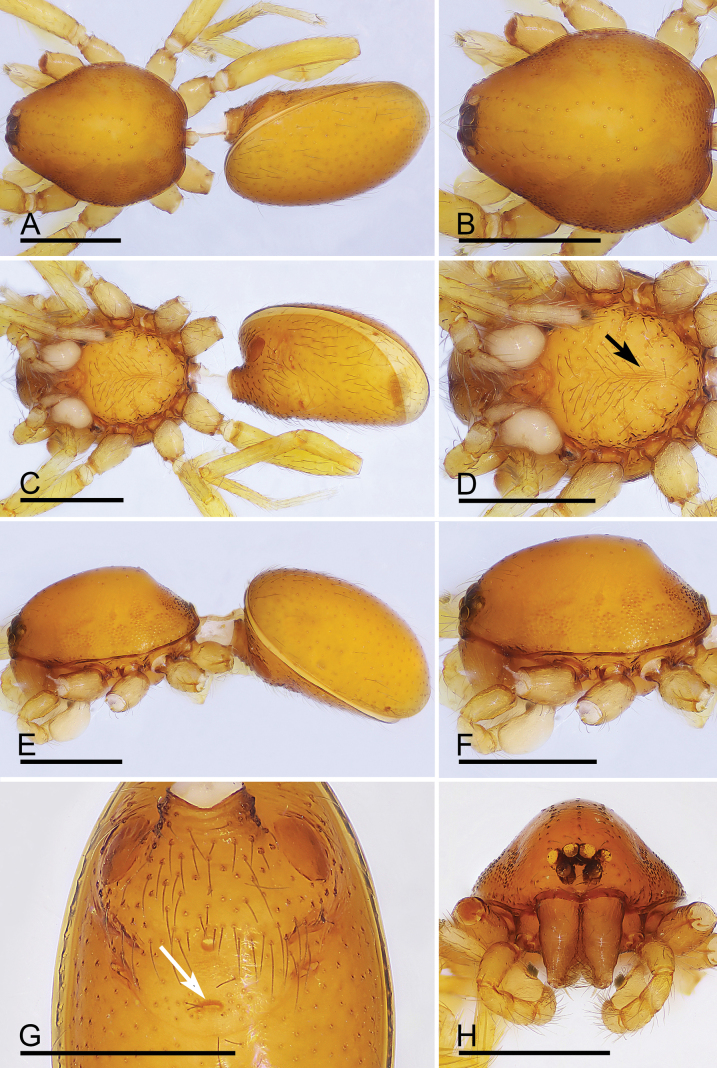
*Trilacunazayu* sp. nov., male holotype **A, C, E** habitus, dorsal, ventral and lateral views **B, D, F, H** prosoma, dorsal, ventral, lateral and anterior views, arrow in **D** shows the small ridges **G** abdomen, ventral view, arrow shows the small slit. Scale bars: 0.4 mm.

**Figure 12.**
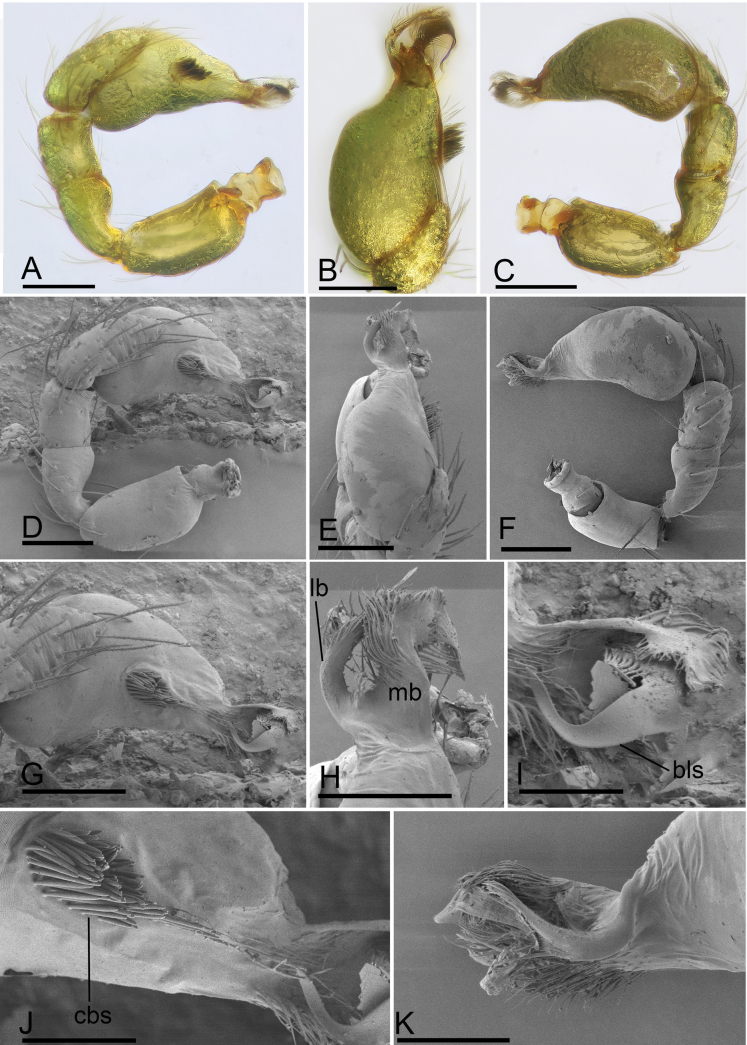
*Trilacunazayu* sp. nov., male left palp **A–C** (light) and **D–K** (SEM) **A, D** prolateral view **B, E** dorsal view **C, F** retrolateral view **G** bulb, prolateral view **H, I, K** distal part of bulb, dorsal, prolateral and retrolateral views **J** detail of bulb, prolateral view. Abbreviations: bls = belt-like structure; cbs = cluster of bristle-like structure; lb = lateral branch; mb = median branch. Scale bars: 0.1 mm (**A–G**); 0.05 mm (**H–K**).

**Figure 13.**
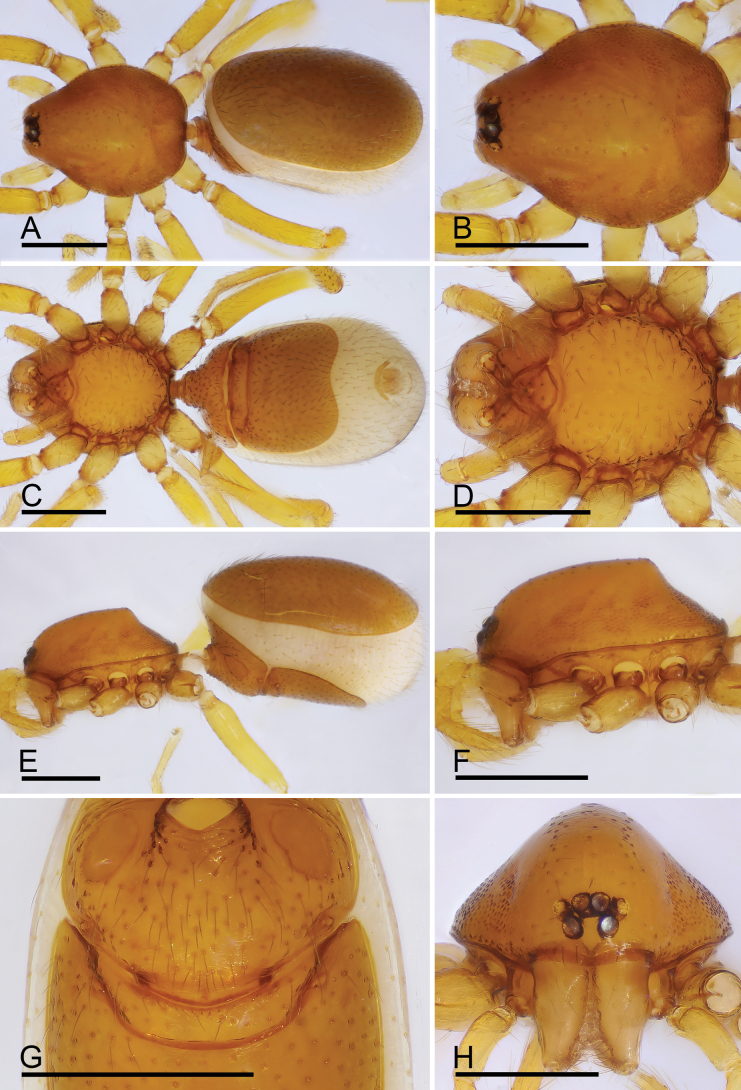
*Trilacunazayu* sp. nov., female paratype **A, C, E** habitus, dorsal, ventral and lateral views **B, D, F, H** prosoma, dorsal, ventral, lateral and anterior views **G** abdomen, ventral view. Scale bars: 0.4 mm.

**Figure 14.**
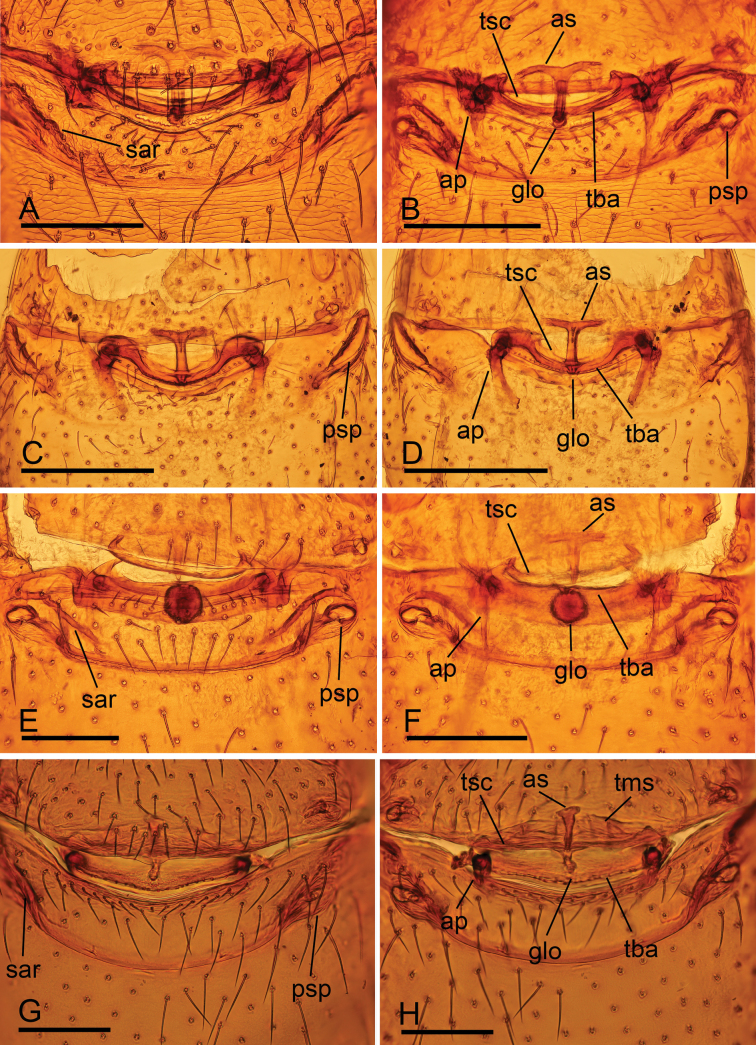
*Trilacuna* spp., endogyne **A, B***Trilacunamainling* sp. nov. **C, D***Trilacunametok* sp. nov. **E, F***Trilacunabangla* Grismado & Ramírez, 2014 **G, H***Trilacunazayu* sp. nov. **A, C, E, G** ventral view **B, D, F, H** dorsal view. Abbreviations: ap = apodeme; as = anterior sclerite; glo = globular structure; psp = posterior spiracle; sar = sclerotized, recurved arches; tba = transverse bars; tms = transverse membranous structure; tsc = transverse sclerite. Scale bars: 0.1 mm.

Figs 5K , 11 , 12 , 13 , 14G, H

#### Material examined.

***Holotype*** China • ♂ (SYNU-908); Xizang, Nyingchi City, Zayu Co., Gaba Oil Field; 28°38.849'N, 97°27.088'E, 2300 m elev.; 25.VII.2013; Q. Cao leg. ***Paratypes*.** China • 1♂ (SYNU-F-928); same data as holotype • 2♀ (SYNU-909–910); Zayu Co., Dongriqugou; 28°39.670'N, 97°28.657'E, 2470 m elev.; 23.VII.2013; L. Lin leg.

#### Other material.

China • 1♂ (SYNU-F-929); Zayu Co., Dongriqugou; 28°39.670'N, 97°28.657'E, 2470 m elev.; 23.VII.2013; Q. Cao leg. • 2♀ (SYNU-F-931–932); same data as above • 1♀ (SYNU-F-930); Zayu Co., Gaba Vill.; 28°40.254'N, 97°27.780'E, 2460 m elev.; 24.VII.2013; L. Lin leg.

#### Etymology.

The specific name is a noun in apposition taken from the type locality.

#### Diagnosis.

The new species is similar to *T.longling* Tong, Zhang & Li, 2019 in the ridges on posterior part of the male sternum, the small slit of the male epigastric region and the T-shaped sclerite of the endogyne, but can be distinguished by the elongated male palpal femur (Fig. 12D) vs. globular (Tong et al. 2019: fig. 13A), the cluster of bristle-like structures (cbs) on the median area of the bulb (Figs 12A, J) vs. lacking (Tong et al. 2019: fig. 13A), the long, belt-like structure (bls) of the psembolus (Fig. 12I) vs. many fiber-like lobes (Tong et al. 2019: fig. 13B), and the transversally elongated membranous structure (tms) of the endogyne (Fig. 14H) vs. lacking (Tong et al. 2019: fig. 24J).

#### Description.

**Male.** Body: yellowish-brown, legs lighter; habitus as in Fig. 11A, C, E; body length 1.64. Carapace (Fig. 11B, F): 0.72 long, 0.58 wide; sides granulate. Eyes (Fig. 11B, F): eyes well developed, nearly equal in size; ALE separated from edge of carapace by 1.4 diameters. Mouthparts (Figs 5K, 11D, H): endites distally branched. Sternum (Fig. 11D): surface smooth; with several rows of ridges in posterior part. Abdomen (Fig. 11A, C, E, G): 0.84 long, 0.49 wide; surface smooth; sperm pore situated at level of anterior spiracles; with small slit anterior of groove connecting posterior spiracles. Palp (Fig. 12A–K): orange; 0.54 long (0.17, 0.10, 0.09, 0.16); femur elongated (width/length = 0.52); bulb triangle, with cluster of bristle-like structure (cbs) on median area; psembolus with long, belt-like structure (bls), a lateral curved branch (lb) and broad median branch (mb).

**Female.** Same as male except as noted. Body: length 1.89; habitus as in Fig. 13A, C, E. Carapace: 0.77 long, 0.63 wide. Abdomen: 1.06 long, 0.67 wide. Epigastric area (Figs 13A, 14G): with recurved, strongly sclerotized arches (sar). Endogyne (Fig. 14H): with narrow, transverse sclerite (tsc) and broad, transversally elongated membranous structure (tms); with anterior slender sclerite (as) and posterior small globular structure (glo); transverse bars (tba) with pair of short lateral apodemes (ap).

#### Distribution.

Known only from the type locality (Fig. 1).

## Supplementary Material

XML Treatment for Trilacuna

XML Treatment for Trilacuna bangla

XML Treatment for Trilacuna mainling

XML Treatment for Trilacuna metok

XML Treatment for Trilacuna zayu

## ﻿Additional information

### Author ORCIDs

Songlu Shi https://orcid.org/0009-0003-6482-9926

Dongju Bian https://orcid.org/0000-0002-9324-8026

Yanfeng Tong https://orcid.org/0000-0002-4348-7029

Shuqiang Li https://orcid.org/0000-0002-3290-5416

### Data availability

All of the data that support the findings of this study are available in the main text.
